# Influence of surface treatments on enamel susceptibility to staining by cigarette smoke

**DOI:** 10.4317/jced.51097

**Published:** 2013-10-01

**Authors:** Juliana C. Públio, Maria BF. D’Arce, Nádia M. Brunharo, Gláucia MB. Ambrosano, Flávio HB. Aguiar, José R. Lovadino, Débora ANL. Lima

**Affiliations:** 1 DDS, MS student. Department of Restorative Dentistry, Piracicaba Dental School, Campinas University – UNICAMP, SP, Brazil; 2 DDS, MS, PhD student. Department of Restorative Dentistry, Piracicaba Dental School, Campinas University – UNICAMP, SP, Brazil; 3 DDS. Department of Restorative Dentistry, Piracicaba Dental School, Campinas University – UNICAMP, SP, Brazil; 4 PhD, Full Professor. Department of Social Dentistry/Statistics, Piracicaba Dental School, Campinas University – UNICAMP, SP, Brazil; 5 DDS, MS, PhD, Assistant Professor. Department of Restorative Dentistry, Piracicaba Dental School, Campinas University – UNICAMP, SP, Brazil; 6 DDS, MS, PhD, Full Professor. Department of Restorative Dentistry, Piracicaba Dental School, Campinas University –UNICAMP, SP, Brazil

## Abstract

Objectives: The purpose of this study was to evaluate the influence of remineralizing agents, including artificial saliva, neutral fluoride, and casein phosphopeptide-amorphous calcium phosphate (CPP-ACP), on the susceptibility of bleached enamel to staining by cigarette smoke. Study Design: Fifty bovine enamel blocks were randomly divided into five groups (n = 10): G1- bleaching; G2- bleaching and immersion in artificial saliva; G3- bleaching and application of CPP-ACP; G4- bleaching and application of neutral fluoride; and G5- untreated (Control). Teeth were bleached with 35% hydrogen peroxide and treated with the appropriate remineralizing agent. After treatment, all groups were exposed to cigarette smoke. Enamel color measurements were performed at three different times: before treatment (T1), after treatment (bleaching and remineralizing agent) (T2), and after staining (T3), by using the CIE Lab method with a spectrophotometer. The data coordinate L* was evaluated by analysis of repeated-measures PROC MIXED and Tukey-Kramer’s test, and the ΔE values were submitted to one-way ANOVA and Tukey’s test (α = 0.05). Results: The G1 group did not show any statistically significant difference for L* values between times T1 and T2. The G4 and G5 groups showed lower L* values at T3 compared to T2. No significant differences between the groups were observed for ΔE (after treatment and staining). However, G4 showed a clinically apparent color change. Conclusions: Treatment of bleached enamel with neutral fluoride can contribute to the increased staining of enamel due to cigarette smoke.

** Key words:**Spectrophotometer, remineralizing agents, bleaching.

## Introduction

Bleaching agents are used to remove intrinsic pigments from dental enamel. Common bleaching techniques include home bleaching (performed by the patient with individual trays) and in-office bleaching (performed by dentists) (1). Options for vital tooth-bleaching procedures include carbamide peroxide and hydrogen peroxide products. The most-accepted theory for the mechanism of action of bleaching agents is the oxidation of pigments in dental structures (2). Bleaching agents are thought to cleave the extensive conjugated chains that comprised the pigments (3), thereby reducing the number of pigment molecules (4).

Several studies have shown that bleached enamel can become stained after exposure to pigments, such as tea, coffee, and red wine (5-8). Some authors consider that the loss of mineral content, which causes decalcification, porosity, and topographic changes (9-11), may favor tooth staining. Attin et al. (11) concluded that mineral loss is compensated for by the remineralization property of saliva, which contains calcium and phosphate ions. Furthermore, the stain absorption is related to the pH, composition, and temperature at which the pigments are exposed.

Although there have been numerous studies of bleaching treatment effectiveness and its adverse effects on tooth enamel and adjacent tissues, there are no reports in the literature about the behavior of newly whitened teeth exposed to cigarette smoke. Thus, the objective of this study was to evaluate the color change of bovine enamel fragments that were bleached with 35% hydrogen peroxide, subjected to different surface treatment protocols (i.e., with saliva, fluoride, or casein phosphopeptide-amorphous calcium phosphate [CPP-ACP]), and then exposed to cigarette smoke.

## Material and Methods

Fifty bovine incisors were stored in 0.1% thymol after collection and disinfection. The teeth were examined under a light microscope (4×) (Carl Zeiss Zeiss-Brazil) for the presence of any irregularities, such as cracks or stains, that would interfere with the research results. If these features were found, then the tooth was discarded and replaced. The teeth were stored in distilled water under cooling until the moment of their use. Fifty dental blocks were extracted from a 5 × 5 mm area of the buccal surface of the bovine incisors through two cuts in the mesio-distal and two cuts in the cervical-incisal directions by using a double-faced diamond disc (EXTEC Diamond Wafering Blade, 102 x 12.7 x 0.3 mm) in a water-cooled diamond saw (IsoMet 1000, Buehler, Lake Bluff, IL, USA). The dentin and enamel surfaces were flattened with #600 and #1200 grit silicon carbide (SiC) paper, in a polishing machine (Arotec Ind. Com., Cotia, SP, Brazil) under constant water irrigation, to obtain a 3-mm-thick block (2 mm of dentin and 1 mm of enamel). In the interval between each application of SiC paper and at the end of the process, the specimens were cleaned with distilled water in an ultrasonic bath (T7 Type, CT Model, Thornton-Inpec electronic Ltd, Vinhedo, SP, Brazil) for debris removal. Each specimen was marked with a diamond bur #1012 (KG Sorensen) on one side, to standardize the sample position in the spectrophotometer (Konica Minolta CM 700d, Japan). The specimens were stored in artificial saliva for 24 hours (12) and maintained at 37 ± 2 °C. Samples were randomly divided into five groups (n = 10), according to the treatment protocol.

- Treatment Protocol

Bleaching: Bleaching with 35% hydrogen peroxide (HP) (Whiteness HP Maxx 35%-FGM, Santa Catarina, Brazil) was performed according to the manufacturer’s instructions. Bleaching agent was applied three times for 15 minutes each to the enamel surface. The specimens were then washed thoroughly in running water.

Surface treatment: After bleaching, one of the following surface treatments was performed to each sample group: Group 1: Samples were submitted to the bleaching protocol and no surface treatment; Group 2 (Artificial Saliva): Samples were immersed in artificial saliva for 30 minutes; Group 3 (CPP-ACP): CPP-ACP paste (MI Paste, GC) was applied to the enamel surface at low speed with a polishing rubber mounted on the handpiece. After 3 minutes, excess paste was removed by air-water spray for 10 seconds; Group 4 (2% Neutral Fluoride): Neutral fluoride was applied to the enamel surface for 4 minutes, and excess fluoride was removed by a cotton-tipped flexible plastic (Swabs, Johnson & Johnson, Brazil); Group 5 (Control): No bleaching or surface treatment was performed; this group was maintained at 37 ± 2 °C in artificial saliva during the experiment.

Exposure to cigarette smoke: Samples were fixed in a device and positioned in a machine to simulate smoke inhalation. The samples remained in contact with the cigarette smoke while cigarettes were burned. Each sample underwent 10 cycles of simulated smoke exposure. The samples were cleaned on each side with a mixture of pumice and water by a polishing rubber mounted at low speed for 30 seconds each side and washed in running water by a single operator. Excess water was removed with absorbent paper before color measurement.

- Color Measurements

Before the color analysis stage was performed for the first time, specimens with similar means were selected and outliers were discarded, to standardize the specimens. Color measurement was performed three times: before bleaching (Initial), after bleaching and surface treatment (After Bleaching), and after exposure to cigarette smoke (After Staining). Specimens were placed in a Teflon device (sample holder) inside a light cabin (GTI Mini Matcher MM1e, GTI Graphic Technology Inc., Newburgh, NY, USA) to standardize the ambient light during the measurement process. The samples were assessed with a previously calibrated spectrophotometer Konica Minolta CM-700d (Konica Minolta Investment Ltd. Sensing Business Division, Shanghai, China), which was used in accordance with the manufacturer’s instructions.

Spectrophotometric values were quantified on the CIE Lab system as three coordinates (L*, a*, b*) that define the color of an object within a three-dimensional color space. A microcomputer with the On Color QC Lite software (Konica Minolta, Japan) was used to generate spectral measurements as a function of wavelength for data-processing and analysis. In the color space, L* indicates lightness (L+ = lightness and L- = darkness), a* coordinate represents the red/green range (a*+ = redness and a*- = greenness) and the b* coordinate represents the yellow/blue range (b*+ = yellowness and b*- = blueness). The values of the coordinates a* and b* approach zero, indicating neutral colors (white and gray) and an increase in magnitude for more saturated or intense colors (3,24). The L*a*b* system provides the numeric definition of a color and the difference between two colors with the following formula: ΔE = [(L1 – L0)2 + (a1 – a0)2 + (b1 – b0)2]1/2.

After exploratory data analysis, the L* variable was subjected to analysis using the methodology of mixed models for repeated-measures by PROC MIXED procedure of SAS statistical software. The treatment means were compared using Tukey-Kramer’s test, considering a 5% significance level. The variables ΔE1, ΔE2 and ΔE3 were explored by one-way analysis of variance (ANOVA) and Tukey’s test at a significance level of 5%.

- Scanning Electron Microscopy (SEM) Analysis

After the bleaching and surface treatments, three samples from each group were randomly selected, dehydrated by immersion in increasing alcohol concentrations, and sputter-coated with gold for SEM analysis (JEOL JSM-5600 LV, Tokyo, Japan).

## Results

- Color Analysis

 Table 1 shows the average (mean and standard deviation) values for L* (L=100- lightness; L= 0- darkness).

**Table 1:**
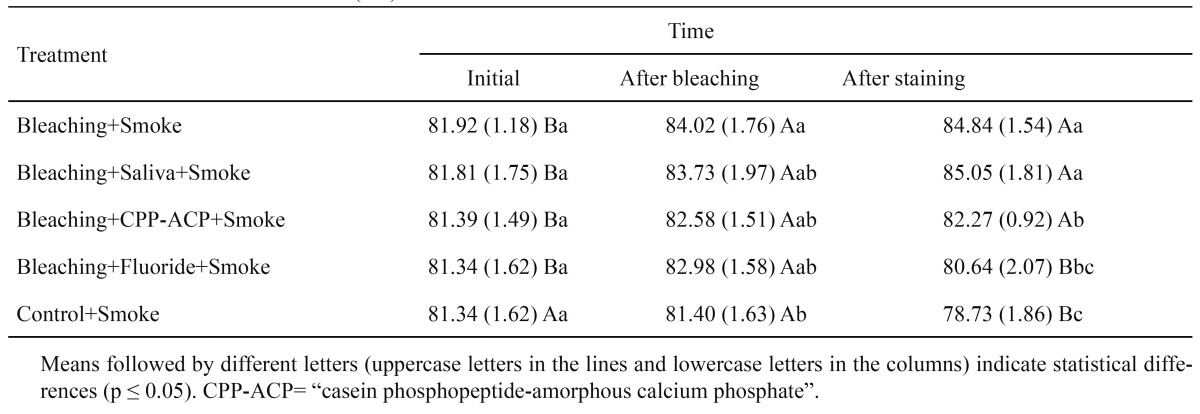
Mean and standard deviation (SD) of L* values as a function of treatment and time.

 Table 2 shows the color variation (ΔE) between treatment sessions: After Bleaching × Initial (ΔE1), After Staining × After Bleaching (ΔE2), and After Staining × Initial (ΔE3).

**Table 2:**
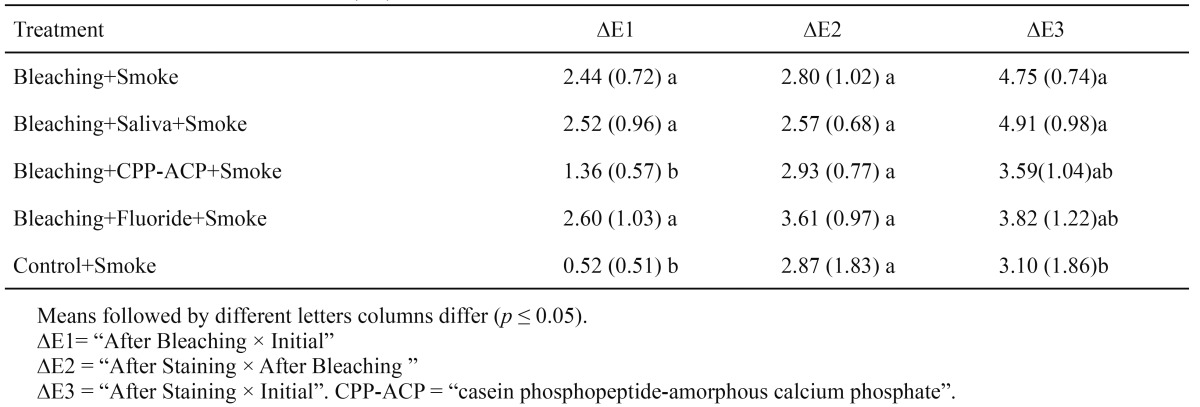
Mean and standard deviation (SD) of ΔE.

- Scanning Electron Microscopy 

**Figure 1:**
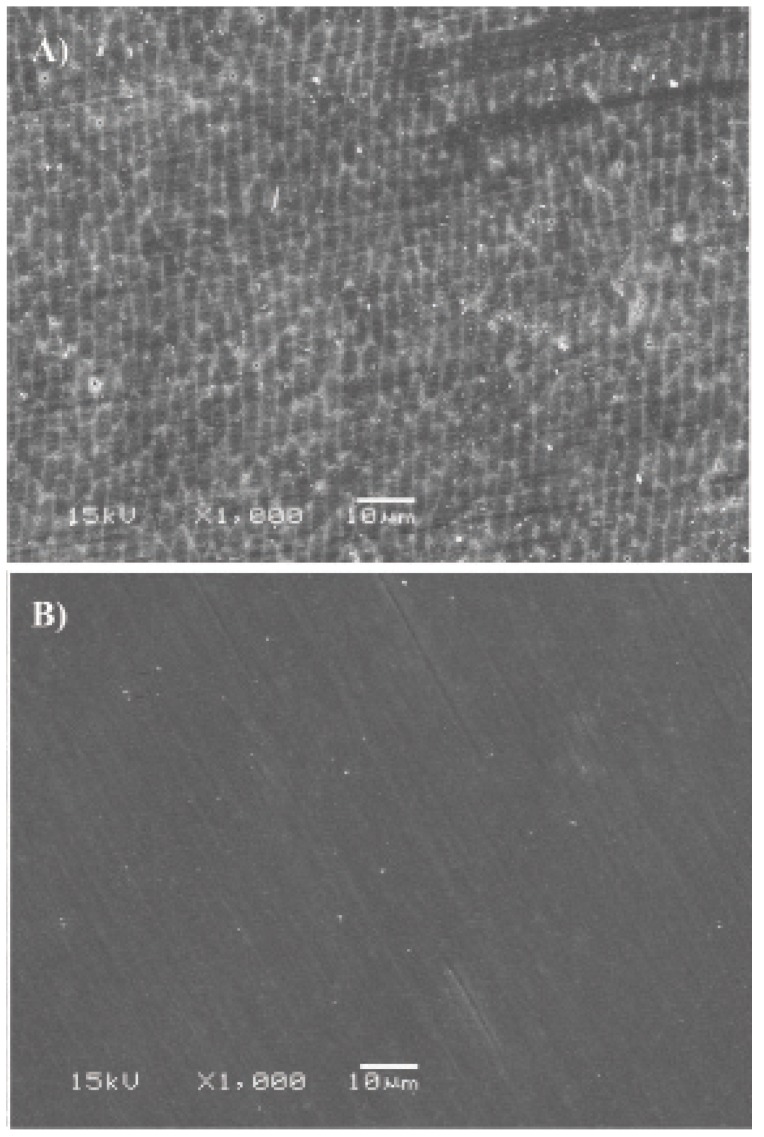
SEM observation: A) Bleaching + Smoke, B) Control + Smoke, at 1000× magnification..

**Figure 2:**
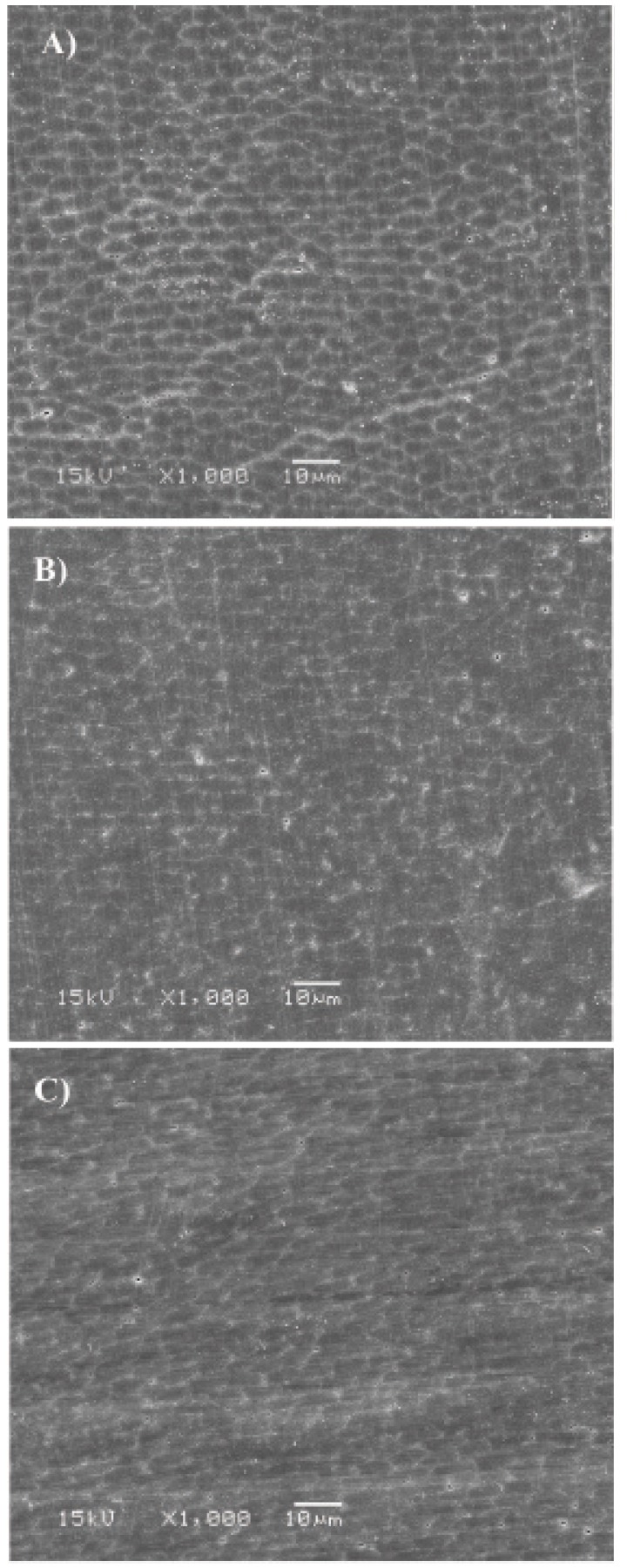
SEM observation: A) Bleaching + Saliva + Smoke; B) Bleaching + CPP-ACP + Smoke; C) Bleaching + Fluoride + Smoke, at 1000× magnification.

Photomicrographs of the surfaces treated with 35% HP (G1) showed changes in the enamel morphology, re-vealing many pores and erosions as well as evidence of enamel rods (Fig. 1), compared to the control group (G5) (Fig. 1). Surfaces treated with artificial saliva (G2) had fewer pores and depressions but evidence of many enamel rods (Fig. 2). Photomicrographs of the CPP-ACP (G3) samples showed slight surface morphological changes (Fig. 2). Surfaces treated with 2% neutral fluoride (G4) (Fig. 2) displayed pores and depressions that were smaller than those of the G1 samples.

## Discussion

In this study, at the After Staining time, the G4 (Bleaching + Fluoride + Smoke) and G5 (Control + Smoke) groups showed the lowest L* means (highest staining), which differed from those at the After Bleaching time. The G1 (Bleaching + Smoke) and G2 (“Bleaching + Saliva + Smoke) groups showed less staining (highest L* means) at the After Staining time compared to the other groups.

Neutral fluoride has been used after bleaching treatment to contribute to enamel remineralization (13). According to Reynolds et al. (14), the capacity of fluoride to promote dental tissue remineralization is linked to the availability of calcium and phosphate ions in the environment. Fluoride ions may guide tissue remineralization if adequate amounts of calcium and phosphate ions are still available in the saliva or dental biofilm (15). Therefore, after fluoride application, the enamel must be exposed to saliva for sufficient time to enable the appropriate saturation of ions to allow for fluorapatite or fluorohydroxyapatite generation on the enamel surface. The microporosities formed after the bleaching treatment and fluoride application were not homogeneously remineralized, which allowed the stain to develop on the enamel surface after cigarette smoke exposure. According to Ferreira et al. (16), fluoride application did not prevent morphological changes on enamel after dental bleaching.

CPP-ACP is another compound that has been used as a remineralizing agent because of its ability to release calcium and phosphate ions in the tooth surface (17). CPP-ACP consists of titanium dioxide, a pigment that is widely used by several industries for its brightness, high refractive index, discoloration resistance (18), and ability to opacify and bleach the environment in which it is dispersed, as described by the manufacturer. This last property of titanium dioxide may have made the enamel surface lighter, which may have interfered with the degree to which the smoke stains were covered up in the final color measurement. In particular, there was no statistical difference for the group treated with CPP-ACP between the after-bleaching and after-staining times, although the CPP-ACP and fluoride groups showed lower L* means when compared to the other groups. The SEM analyses of surfaces bleached and treated with CPP-ACP paste are consistent with the findings of Cunha et al. (19), who also observed small irregularities on the enamel surface.

The “Bleaching” (G1) and “Saliva + Bleaching” (G2) groups showed the highest L* means compared to the other groups. The ΔE means for these two groups, comparing the overall color change between “After Staining × Initial” (ΔE3) in  Table 2, were also the highest means showing a statistically significant result, because the results with the CPP-ACP and fluoride treatments did not differ from the final means. According to Cavalli et al. (20), as a remineralizing solution, saliva repairs the tooth surface microstructures through the absorption and precipitation of salivary calcium and phosphate ions. In an in vitro study, Liporoni et al. (21) also observed that storage in artificial saliva can promote a lower amount of staining of the dental surface, due to its remineralizing potential.

The photomicrographs of enamel treated with 35% HP revealed an irregular pattern in the exposure of enamel rods (Fig. 1). This microporosity of bleached enamel is caused by the degradation of the organic material (22,23), which is independent of the gel pH (24). Ferreira et al. (16) observed by SEM that a 35% HP bleaching agent causes porosity, irregularities, and depressions on the tooth surface. Although these superficial changes are clinically imperceptible (25), several studies have shown that they can favor pigment accumulation that would interfere with the longevity of dental bleaching. In this study, the enamel surfaces were subjected to prophylaxis after cigarette smoke exposure, to simulate the clinical condition. Prophylaxis may have led to the removal of the demineralized area and, possibly, the pigmented enamel, which could explain why the bleached surface was not pigmented after smoke exposure.

The CPP-ACP and neutral fluoride treatments did not prevent the accumulation of pigments on remineralized enamel surfaces when these surfaces were exposed to cigarette smoke. Even though CPP-ACP promotes the absorption and precipitation of calcium and phosphate ions, deposition of these ions can occur in an irregular way and might increase the susceptibility to enamel staining. In an in vitro study, Singh et al. (26) observed that CPP-ACP and topical fluoride surface treatments prevented bleached enamel staining. However, their samples were stored in artificial saliva after surface treatment, which may explain how this result was achieved.

In conclusion, the results of this study show that bleached enamel exposed to artificial saliva for 30 minutes shows the lowest level of staining by cigarette smoke, and the treatment of bleached enamel with neutral fluoride can contribute to increase enamel staining due to cigarette smoke. Thus, after in-office dental bleaching treatment is performed for smokers, the patient should wait at least 30 minutes before smoking, to allow for the remineralization of the enamel by saliva and to avoid staining the tooth enamel.
